# Cell Adhesion and *in Vivo* Osseointegration of Sandblasted/Acid Etched/Anodized Dental Implants

**DOI:** 10.3390/ijms160510324

**Published:** 2015-05-06

**Authors:** Mu-Hyon Kim, Kyeongsoon Park, Kyung-Hee Choi, Soo-Hong Kim, Se Eun Kim, Chang-Mo Jeong, Jung-Bo Huh

**Affiliations:** 1 Department of Prosthodontics, Dental Research Institute, Biomedical Research Institute, School of Dentistry, Pusan National University, Yangsan 676-870, Korea; E-Mails: lohasdent@gmail.com (M.-H.K.); cmjeong@pusan.ac.kr (C.-M.J.); 2 Division of Bioimaging, Chuncheon Center, Korea Basic Science Institute, Gangwon-do 200-701, Korea; E-Mail: kspark1223@hanmail.net; 3 Research Development Institute, Cowellmedi Co., Ltd., Busan 617-801, Korea; E-Mails: ckh@cowellmedi.com (K.-H.C.); ksh@cowellmedi.com (S.-H.K.); 4 Department of Veterinary Surgery, College of Veterinary Medicine, Chonnam National University, Gwangju 500-757, Korea; E-Mail: sen0223@gmail.com

**Keywords:** titanium implant, sandblasting and acid etching, anodization, osteoblast, osseointegration

## Abstract

The authors describe a new type of titanium (Ti) implant as a Modi-anodized (ANO) Ti implant, the surface of which was treated by sandblasting, acid etching (SLA), and anodized techniques. The aim of the present study was to evaluate the adhesion of MG-63 cells to Modi-ANO surface treated Ti *in vitro* and to investigate its osseointegration characteristics *in vivo*. Four different types of Ti implants were examined, that is, machined Ti (control), SLA, anodized, and Modi-ANO Ti. In the cell adhesion study, Modi-ANO Ti showed higher initial MG-63 cell adhesion and induced greater filopodia growth than other groups. *In vivo* study in a beagle model revealed the bone-to-implant contact (BIC) of Modi-ANO Ti (74.20% ± 10.89%) was much greater than those of machined (33.58% ± 8.63%), SLA (58.47% ± 12.89), or ANO Ti (59.62% ± 18.30%). In conclusion, this study demonstrates that Modi-ANO Ti implants produced by sandblasting, acid etching, and anodizing improve cell adhesion and bone ongrowth as compared with machined, SLA, or ANO Ti implants. These findings suggest that the application of Modi-ANO surface treatment could improve the osseointegration of dental implant.

## 1. Introduction

Osseointegration, defined as intimate contact between living bone and implant surface, is considered a prerequisite for implant loading and the long-term clinical success of dental implants [1,2,3]. Pure titanium (Ti) and its alloys are frequently used in dental and orthopedic implants because they have excellent mechanical strength, chemical stability and biocompatibility [4]. However, poor binding of pure Ti implants to bone cells and tissues extends the required time between surgery and implant loading, and ultimately leads to implant failure [3]. Therefore, the enhancement of osseointegration presents a continued challenge to those designing Ti dental implants [5,6,7].

Much research has been focused on the modification of implant surface properties that substantively determine osseointegration during bone healing. Various surface treatment techniques, such as sandblasting, acid-etching, grit-blasting, anodization, plasma-spraying, coating with inorganic calcium phosphate or biological molecules, and chemical modifications have been devised to improve biological characteristics that promote osseointegration and bone formation, and thus, shorten the time require to implant loading [8,9,10,11,12]. Among these surface modification techniques, sandblasting with large grit media with acid etching (SLA) and anodizing (ANO) are frequently used to modify the surfaces of Ti dental implant. SLA treatment involves blasting implant surface with large grit particles (250~500 μm) and subsequently acid-etching the implant surface [13]. The improved surface roughnesses obtained after SLA treatment enhance the osteoconductive process, bone-to-implant contact, and increase removal torque [14]. The anodized surface treatment is an electrochemical process based on anodic oxidation, which alters the surface topography and chemical composition of the surface oxide layer [15]. The Ti oxide layer and micropores formed by anodization improve the cellular activities (e.g., cell adhesion and proliferation) and enhance osseointegration *in vivo* [16,17].

These surface treatments have contributed to the clinical performance of dental implants. However, there remains a need to shorten times to healing and loading. To do this, several new approaches have been developed by combining or modifying existing surface treatment techniques. For example, combined surface treatment by blasting with resorbable large grit media and anodization has been tried. In this study, we fabricated a new type of surface modified Ti implant (termed Modi-ANO) by combining two surface modification techniques namely sandblasting/acid-etching (SLA) and anodizing (ANO). Also, we investigated the osseointegration of the Modi-ANO implants *in vitro* and *in vivo* in a beagle model.

## 2. Results

### 2.1. Surface Characterization

**Figure 1:**
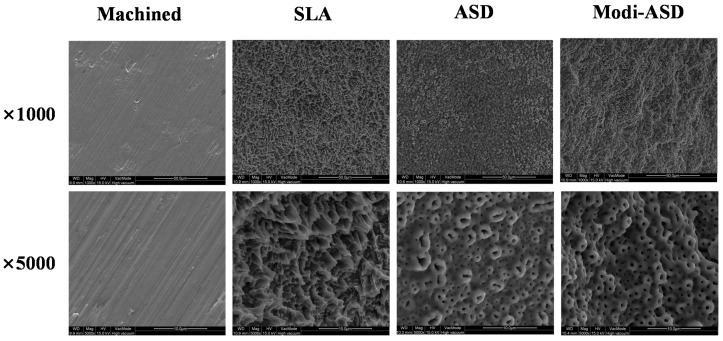
Scanning electron microscopy (SEM) images of surface-modified titanium substrates. SLA: sandblasted and acid etched Ti, ANO: anodized Ti, Modi-ANO: sandblasted/acid etched and anodized Ti.

The surface topographies of sandblasted and acid etched Ti (SLA), anodized Ti (ANO), and sandblasted/acid etched and anodized Ti (Modi-ANO) were determined using the scanning electron microscopy (SEM) (Figure 1). Control discs had a smooth surface with slight scratches caused by mechanical polishing. The surfaces of SLA discs were rough and irregular and needle-like elevations were also observed. The microroughness of SLA discs was 2–4 μm. ANO discs had a porous structure (pores resembled the craters of volcanoes) induced by anodic oxidation of pore size 0.5–3.0 μm. Modi-ANO discs had a surface morphology and pore size similar to that of ANO discs, but were rougher and more irregular. Qualitative and quantitative roughness values are given in Table 1. Consistent with SEM results, the order of roughness was SLA > Modi-ANO > ANO > control. EDS-surface analysis revealed the presence of essentially Ti surfaces for all four finishes. However, additional elements, such as, phosphorous, and oxygen were found on the surfaces of ANO and Modi-ANO groups (Table 2).

**Table 1:** Surface roughnesses of the titanium substrates as determined by stereo scanning electron microscopy.

Groups	Sa (μm) ± SD	Sq (μm) ± SD	Rt (μm) ± SD
**Control**	0.08 ± 0.04	0.09 ± 0.02	1.95 ± 0.11
**SLA**	1.68 ± 0.22	2.15 ± 0.29	16.99 ± 3.09
**ANO**	0.52 ± 0.14	0.68 ± 0.19	6.44 ± 1.51
**Modi-ANO**	1.45 ± 0.25	1.85 ± 0.33	13.76 ± 2.69

Sa: roughness per unit surface: the average of absolute values of the surface protrusion on the mean surface; Sq: the root mean squared values of the protruding surface from the mean surface; Rt: distances from the line of the maximum and minimum heights of the protruding surface.

**Table 2:** EDS surface elemental analysis results.

Groups	C (at%)	O (at%)	P (at%)	Ti (at%)
**Control**	7.38	15.71	0.96	75.95
**SLA**	–	3.4	–	96.6
**ANO**	5.35	65.20	5.74	23.71
**Modi-ANO**	5.76	69.24	5.61	19.39

### 2.2. Cell Adhesion Testing

**Figure 2:**
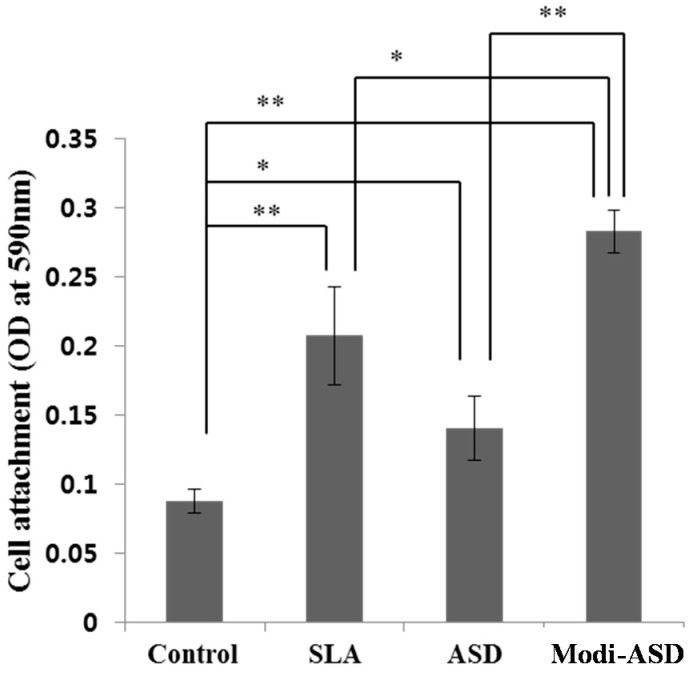
Cell adhesion of MG-63 cells cultured on titanium substrates. Crystal violet assays were performed after incubating MG-63 cells on each Ti surface for 3 h. * p < 0.05 and ** p < 0.01 indicate statistical significant between groups.

Initial cell adhesions for MG-63 cells on Ti substrates *versus* the control group were 2.4-, 1.53-, and 3.18-fold higher for the SLA, ANO, and Modi-ANO groups (Figure 2). Interestingly, the Modi-ANO group showed higher initial cell adhesion than the SLA and ANO groups (* *p* < 0.05).

**Figure 3:**
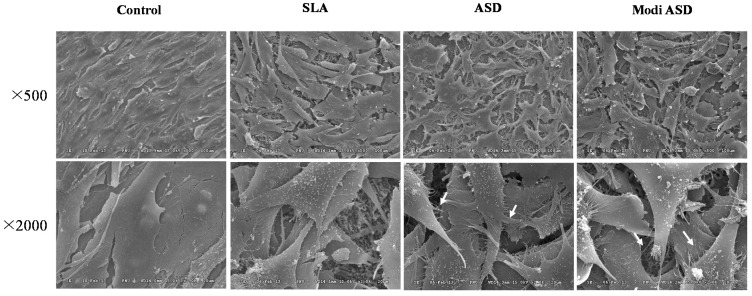
SEM images of the morphologies of MG-63 cells grown on Ti control, SLA, ANO, and Modi-ANO titanium substrates for 24 h.

The morphology of MG-63 cells cultured on control, SLA, ANO, and Modi-ANO Ti substrates were studied by SEM after 24 h of incubation (Figure 3). MG-63 cells actively adhered to SLA, ANO, and Modi-ANO Ti substrates after 24 h of incubation. Numerous filopodia, which extended in all directions, were observed in the ANO and Modi-ANO groups.

### 2.3. In Vivo Study

**Figure 4:**
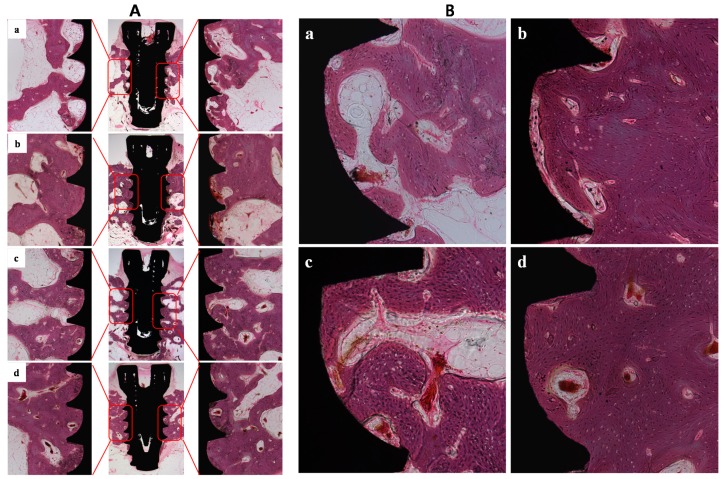
(**A**) Histological evaluations of (**a**) Control; (**b**) SLA; (**c**) ANO; and (**d**) Modi-ANO. (Magnification: Central, ×12.5; Right and Left, ×40); (**B**) Histological images of (**a**) Control; (**b**) SLA; (**c**) ANO; and (**d**) Modi-ANO (Original magnification ×100).

Eight weeks after Ti-implant surgery, histological examinations were performed to evaluate bone formation, bone-to-implant contact (BIC), and intra-thread bone density (ITBD) for each of the inserted implant types. Bone formation on the implants was analyzed at the portion of the upper three threads as shown in Figure 4. Compared to controls, SLA, ANO and Modi-ANO implants showed enhanced osseointegration, that is, more mature bone formation was observed on implant surfaces. Table 3 shows that the BIC values of the SLA, ANO, and Modi-ANO implants were significantly greater than those of control implants (** *p* < 0.01). In particular, BIC values of Modi-ANO were much higher than those of SLA and ANO (* *p* < 0.05). ITBD values of SLA, ANO and Modi-ANO implants were also higher than control values (** *p* < 0.01), and although the ITBD values of ANO and Modi-ANO were higher than those of SLA, no significant difference was observed. Taken together, these results indicate that the SLA, ANO, and Modi-ANO surface finishes increased osseointegration and bone formation, and that Modi-ANO implants exhibit better osseointegration and bone formation.

**Table 3:** BIC and ITBC values of the four surface treatment types.

Group	Number	BIC (Mean ± SD) (%)	ITBD (Mean ± SD) (%)
**Control**	8	33.58 ± 8.63 **	34.90 ± 7.24 **
**SLA**	8	58.47 ± 12.89 *	53.98 ± 13.77
**ANO**	8	59.62 ± 18.30 *	61.64 ± 16.17
**Modi-ANO**	8	74.20 ± 10.89	60.80 ± 13.32

BIC (%): bone-to-implant contact ratio in the second and third uppermost threads; ITBD (%): intra-thread bone density in the second and third uppermost threads; * p < 0.05 and ** p < 0.01 indicate significantly different from Modi-ANO implants.

## 3. Discussion

Osseointegration of Ti implant surfaces is strongly influenced by surface physical and chemical properties, such as, surface chemistry, charge, topography, wettability, and roughness [18]. In the present study, we developed a new type of surface modified Ti implant (Modi-ANO) by combining sandblasting/acid-etching (SLA) and anodizing (ANO), and evaluated its effects on MG-63 cell adhesion and osseointegration in a dog model.

The surface properties of dental implants play a key role in the biological interactions between implant surfaces and host bone. In particular, it has been demonstrated that surface modifications of dental implants, such as, surface topography, chemical composition, and roughness, that influence osseointegration increase the adhesion and differentiation of osteoblastic cells on implant surfaces [19,20,21], bone-to-implant contact, and biomechanical interactions during the early period post-implantation [22], and ultimately improve implantation success rates. Commercially machined Ti commonly used for dental implants and has a relatively smooth surface. However, as was shown in an *in vitro* study, osteoblasts may not adhere sufficiently or differentiate well on machined Ti. In addition, in an *in vivo* study, it was shown that bone-to-implant contact for machined Ti is unsatisfactory *in vivo*. In the present study, On SLA, ANO and Modi-ANO surfaces, numbers of adherent osteoblastic cells were significantly greater than on machined Ti, and adherent cells were more spread out and formed numerous filopodia in all directions. *In vivo* histological and quantitative results revealed that bone-to-implant contact (BIC) and intra-thread bone density (ITBD) values were significantly higher for SLA, ANO, and Modi-ANO than for the Ti control. Interestingly, BIC values for Modi-ANO were higher than those of SLA and ANO. Many investigators have shown that surface properties, such as, topography, chemical composition, and roughness can significantly alter osseointegration because cells react differently to such surfaces. Furthermore, previous *in vitro* and *in vivo* studies have demonstrated that surface modification of Ti implants can enhance osseointegration [18,23,24,25,26,27,28]. The increased cell attachment and BIC and bone density values observed for SLA, ANO and Modi-ANO implants can be explained as follows. Anodization is an electrochemical process that increases surface roughness and the thickness of the Ti oxide layer, and this layer increases the wettability and biocompatibility of surfaces and ultimately improves osteoblast attachment, proliferation, and bone response [29,30]. Sandblasting/acid-etching has been previously reported to roughen surfaces and to increase wettability and microtexture, and thus, bone integration [31,32]. In the present study, the use of tricalcium phosphate nanoparticles during the sandblasting process created a biomimetic calcium phosphate coating layer on Ti surfaces, which may have improved osteoconductivity [33]. Taking into consideration the above, it appears SLA and/or ANO surface treatments increased the surface adhesion of osteoblasts and enhanced osseointegration and bone formation. In particular, Modi-ANO treatment resulted in better *in vitro* cell adhesion, more filopodia, and higher BIC and/or bone density values than SLA or ANO. We believe that the enhanced effects observed for the Modi-ANO combined treatment were due to advantages conferred by the two surface treatment types. Therefore, we suggest that combined surface treatment using SLA and ANO techniques offers a potential means of improve implant osseointegration.

## 4. Experimental Section

### 4.1. Materials

In the present study, two types of Ti substrates were used, that is, a disc (thickness = 3 mm, diameter = 10 mm) for *in vitro* studies and a grade 4 implant type (length = 7 mm, diameter = 3.75 mm; Cowellmedi Co., Busan, Korea) for the animal study. Four surface treatments were applied to the two Ti substrates, that is, machined Ti (the control group), sandblasted and acid etched Ti (the SLA group), anodized Ti (the ANO group), and sandblasted/acid etched and anodized Ti (the Modi-ANO group). Surfaces in the SLA group were prepared by sandblasting using tricalcium phosphate particles (0.18~0.5 mm diameter) onto machined Ti surfaces, and then acid-etching using HCl:H_2_PO_4_:H_2_O (2:1:1, *v*/*v*/*v*) at 80 °C for 1 h. Surfaces in the ANO group were prepared in an electrochemical cell byanodizing. The Ti substrate was made anode and a platinum rod was used as cathode. The ANO procedure was performed in aqueous electrolyte containing 5% H_2_SO_4_ and 3% H_3_PO_4_ at 180 V for 3 min. Modi-ANO surfaces were prepared by sandblasting and acid-etching as described for the SLA group, and then anodizing using the same protocol used for the ANO group.

### 4.2. Surface Characterization by Scanning Electron Microscopy (SEM)

Ti disc samples were platinum-coated using a sputter coater (SCD 005, BAL-TEC (Leica Microsystems GmbH), Wetzlar, Germany), and surface morphologies were observed under a field emission scanning electron microscope (FE-SEM; Quanta 200, FEI, Hillsboro, OR, USA). In addition, energy-dispersive spectrometric analysis (EDS, Quanta 200, FEI, Hillsboro, OR, USA) was performed to identify the surface components.

### 4.3. Surface Roughness Measurement

The surface roughnesses of samples were determined using 3D surface images (×800 magnification) taken at 6° using a stereo scanning electron microscope (Zeiss EVO25; Zeiss, Germany) and MeX V5.1 software (Alicona, Grambach, Austria). The following were calculated using a Gaussian filter with a cutoff wavelength of kc = 31 µm on surface areas of 340 × 250 µm^2^; Sa (average absolute value of surface protrusions on the mean surface), Sq (root mean squared value of the protruding surface from the mean surface) and Rt (the average distance from the line of the maximum and minimum heights of the protruding surface).

### 4.4. Cell Culture and Cell Adhesion Study

MG-63 cells (osteoblast-like cells) were obtained from the Korean Cell Line Bank (Seoul, Korea) and cultured in Dulbecco’s minimal essential media (DMEM, PAA Laboratories GmbH, Pasching, Austria) containing 10% FBS (PAA) and 1% penicillin-streptomycin (PAA), at 37 °C in humidified 5% CO_2_. In all cell experiments, MG-63 cells were cultured in differentiation inducing media (containing 50 μg/mL ascorbic acid (Sigma-Aldrich, St. Louis, MO, USA), 10 mM β-glycerophosphate (Sigma-Aldrich), 100 nM dexamethasone (Sigma-Aldrich) and 10% FBS and 1% penicillin-streptomycin).

To evaluate cell adhesion on each Ti substrate, MG-63 cells were seeded at a density of 4 × 10^4^ cells/mL on each substrate and incubated at 37 °C for 3 h. Adherent cells were stained with 0.2% crystal violet (Sigma-Aldrich) in 10% ethanol and left at room temperature for 5 min. After washing off remaining dye with PBS, stained cells were dissolved for 15 min and carefully transferred into a 96-well plate. Absorbances were measured at 590 nm using an enzyme-linked immune sorbent assay (ELISA) analyzer (Quant, Bio-Tek, Highland Park, NJ, USA).

Adhesion patterns of MG-63 cells on Ti substrates were observed by SEM (HITACHI S3500N, Japan). MG-63 cells (2 × 10^4^ cells/well) were cultured in a 24-well plate containing Ti substrate at 37 °C for 1 day, and substrates were then rinsed three times with PBS (pH 7.4) and fixed with 2.5% glutaraldehyde for 20 min. After secondary fixation with 1% osmium tetroxide, cells were dehydrated in a gradual ethanol/distilled water series from 70% to 100% in steps of 10% at 4 °C for 10 min each, and lyophilized for 1 day. Specimens were then coated with Au using a sputter-coater, and examined at an accelerating voltage of 15 kV.

### 4.5. In Vivo Animal Study

#### 4.5.1. Fabrication of Implants and Animals

A total of 32 Ti implants (length = 7 mm, diameter = 3.75 mm; Cowellmedi Co., Busan, Korea) of the screw-type were also divided into four groups as described for the Ti disc samples. Each group contained 8 implants and implants used in each group were fabricated with the same methods as described in materials part.

Rearing, management, and surgical procedures were approved by the Animal Ethics Committee of Chonnam National University (Approval No. CNU IACUC-YB-2010-10). Four beagle dogs (1~2 years old, 15 kg) were reared in individual cages in a controlled environment (20–25 °C, RH 40%–60%). Animals were fed a soft-dog food diet (Science Diet, Hill’s Pet Co., Topeka, KS, USA) and had free access to water.

#### 4.5.2. Surgery for Tooth Extraction

Animals were pre-anaesthetized with atropine sulfate (0.05 mg/kg intramuscular (IM) injection; Dai Han Pharm Co., Seoul, Korea) and tiletamine/zolazepam (5 mg/kg IV; Zoletil 50; Virbac, Carros, France) and anesthesia was maintained with isoflurane (Choongwae Co., Seoul, Korea) and oxygen. Local anesthesia was achieved by infiltrating 1 mL of lidocaine (Yu-Han Co., Gunpo, Korea) containing epinephrine (1:100,000) into mucosa at surgical sites. All mandibular premolars and first molars were separated into mesial and distal roots. Care was taken to preserve the buccal, lingual, and lateral walls of alveolar sockets. Teeth were carefully extracted without damaging extraction sockets. Extraction sites were sutured with 4-0 nylon (Mersilk, Ethicon Co., Livingston, West Lothian, UK) to enhance healing. After tooth extraction, meloxicam (Metacam, 0.1 mg/kg PO; Boehringer Ingemheim Co., Ridgefield, CT, USA) was administered for pain relief, and amoxicillin (20 mg/kg PO; JW Pharmaceutical, Seoul, Korea) was administered 12 hourly for 6 days. Extraction sites were allowed to heal for 2 months.

#### 4.5.3. Surgery for Implant Insertion and Postoperative Care

**Figure 5:**
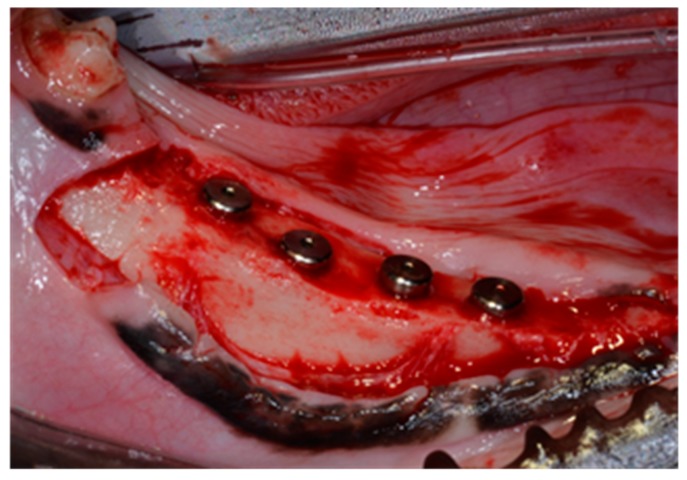
Implant placement. Implants were placed 4 mm apart using a ruler. Releasing incision was made for tension free structure of the flap.

Two months after tooth extraction when healing was complete, experimental implants were installed into the edentulated mandibular alveolar ridge. General and local anesthesia procedures were performed as described above for tooth extraction. The alveolar ridge was trimmed about 1.5 mm to make a flat ridge before inserting implants, and a vertical defect allowing 2.5 mm of the upper portion of implants to be exposed was formed (Figure 5). Each of the four types of experimental implants was installed on the right and left edentulated mandibular alveolar ridge area using a split-mouth design. Treatments were randomized between left and right jaw quadrants in consecutive animals. The installed implants were placed at interval of 4.5 mm (Figure 5). Exposed bone was marked at implant placement sites using a ruler to enable implants to be placed at same locations on both sides. Cefazolan (20 mg/kg) was intravenously injected immediately and at 48 h after surgery. Plaque control was maintained by daily flushing of the oral cavity with 2% chlorohexidine gluconate until study completion. Mucosal health, maintenance of suture line closure, edema, and evidence of tissue necrosis or infection were monitored daily. Suture materials were removed 1 week after implant placement. The animals were given a soft diet for 2 weeks, followed by a conventional regular diet. At 8 weeks post-surgery animals were anesthetized and euthanized by administering concentrated sodium pentobarbital IV (Euthasol, Delmarva Laboratories Inc., Midlothian, VA, USA). Following euthanasia, block sections were collected of implants, alveolar bone, and surrounding mucosa.

#### 4.5.4. Animal Sacrifice and Histological Analysis

At 8 weeks after implant surgery, animals were sedated with intramuscular zaperone (Bayer Animal Health, Isando, South Africa) and midazolam (1 mg/kg), and then sacrificed with an IV injection of 20% pentobarbital solution (Dermocal AG, Buenos Aires, Argentina).

The tissue specimens including implants were prepared, fixed using neutral buffered formalin solution (Sigma-Aldrich) for 2 weeks, and dehydrated in ascending concentrations of ethanol (70%, 80%, 90% and 100%). Dehydrated specimens were embedded in Technovit 7200 resin (Heraeus KULZER, South Bend, IN, USA) and longitudinally sectioned from the center of each implant using an EXAKT diamond cutter (KULZER EXAKT 300, EXAKT, Norderstedt, Germany). Tissue slides (30 μm) were prepared from the initial 400 μm slides by grinding using an EXAKT grinding machine (KULZER EXAKT 400CS, EXAKT), and stained with hematoxylin and eosin. Images were captured using a light microscope (Olympus BX, Tokyo, Japan) equipped with a computer and connected to a CCD camera (Polaroid DMC2 digital Microscope camera; Polaroid Corp., Cambridge, MA, USA). All measurements were made using an image analysis computer program (Image-Pro Plus™, Media Cybernetic, Silver Spring, MD, USA).

The following factors were assessed: (1) Bone-to implant contact (BIC) in the three upper threads. After measuring the total length of the three upper threads, bone-to-implant contact ratio (%) was calculated by measuring the length of bone contact; (2) Intra-thread bone density (ITBD) in the three upper threads. The total intra-thread area between the three upper threads was measured. Bone density (%) was calculated by measuring the area occupied by bone.

### 4.6. Statistical Analysis

Statistical comparisons for cell studies were carried out using one-way ANOVA and SPSS ver. 18.0 (SPSS Inc., Chicago, IL, USA), and then multiple comparisons were performed. For *in vivo* animal studies, the significances of differences between groups were determined using the Kruskal–Wallis test. When the Kruskal–Wallis test indicated differences were significant, multiple comparison analysis was performed as a *post hoc* test using the Mann–Whitney U test. *p* values of <0.05 or <0.01 (* *p* or ** p, respectively) were deemed significant.

## 5. Conclusions

We developed new type of surface modified Ti implant (Modi-ANO) using a combination of SLA and ANO surface treatments. As compared with the Ti control, SLA, ANO, and Modi-ANO implants enhanced osteoblast adhesion and filopodia formation. Also, Modi-ANO treatment showed better osseointegration and bone formation than another groups because it produced more bone-to-implant contact and greater intra-thread bone density *in vivo*. These results indicate that the Modi-ANO surface finishing technique for titanium could improve osseointegration and bone formation after dental implantation.
